# Supplementary material for: CXCR6+ Tumor-Associated Macrophages Identify Immunosuppressive Colon Cancer Patients with Poor Prognosis but Favorable Response to Adjuvant Chemotherapy
Source: Cancers (Basel). 2022 Sep 24;14(19):4646. doi: 10.3390/cancers14194646 (PMC9562861; doi:10.3390/cancers14194646)
Supplement: Supplementary file 1 [file cancers-14-04646-s001.zip › cancers-1900742-supplementary.pdf]

Supplementary Table S1. Baseline of clinicopathological characteristics of cohorts

	Primary cohort	Validation cohort
**Factors**	**No (%)**	**No (%)**
**All patients**	360 (100)	126 (100)
**Gender**		
Female	140 (38.9)	50 (39.7)
Male	220 (61.1)	76 (60.3)
**Age**		
≤60	185 (51.4)	68 (54.0)
＞60	175 (48.6)	58 (46.0)
**Preoperative serum CEA（ng/ml)**		
≤5	194 (53.9)	66 (52.4)
＞5	166 (46.1)	60 (47.6)
**Primary tumor location**		
Right-sided colon	216 (60.0)	65 (51.6)
Left-sided colon	144 (40.0)	61 (48.4)
**Primary tumor size（cm）**		
≤4	200 (55.6)	53 (42.1)
＞4	160 (44.4)	73 (57.9)
**Histology**		
Non-mucinous	314 (87.2)	103 (81.7)
Mucinous	46 (12.8)	23 (18.3)
**Primary Differentiation**		
Well/moderately	259 (71.9)	93 (73.8)
Poorly/undifferentiated	101 (28.1)	33 (26.2)
**T stage**		
T1	14 (3.9)	6 (4.8)
T2	135 (37.5)	47 (37.3)
T3	123 (34.2)	48 (38.1)
T4	88 (24.4)	25 (19.8)
**N stage**		
N0	179 (49.8)	64 (50.8)
N1	120 (33.3)	47 (37.3)
N2	61 (16.9)	15 (11.9)
**M stage**		
M0	263 (73.1)	82 (65.1)
M1	97 (26.9)	44 (34.9)
**TNM stage**		
I	55 (15.3)	20 (15.9)
II	99 (27.5)	25 (19.8)
III	109 (30.3)	35 (27.8)
IV	97 (26.9)	44 (34.9)
**Nerve invasion**		
No	331 (91.9)	118 (93.7)

Yes	29 (8.1)	8 (6.3)
**Vascular invasion**		
No	311 (86.4)	110 (87.3)
Yes	49 (13.6)	16 (12.7)
**Lymph nodes examined**		
＜12	29 (8.1)	9 (7.1)
⩾12	331 (91.9)	117 (92.9)
**MMR status**		
pMMR	321 (89.2)	110 (87.3)
dMMR	39 (10.8)	16 (12.7)

CEA, carcinoembryonic antigen; MMR, mismatch repair.

Supplementary Table S2. Cox regression analysis for OS of validation cohort

	Univariate		Multivariate	
	HR（95% CI）	*p*	HR（95% CI）	*p*
**Gender**				
Female	1 (reference)			
Male	0.73 (0.40-1.33)	0.299		
**Age**				
≤60	1 (reference)			
＞60	0.77 (0.42-1.43)	0.412		
**Preoperative serum CEA （ng/ml)**				
≤5	1 (reference)			
＞5	1.65 (0.90-3.03)	0.106		
**Primary tumor location**				
Right-sided colon	1 (reference)			
Left-sided colon	0.92 (0.50-1.69)	0.800		
**Primary tumor size（cm）**				
≤4	1 (reference)			
＞4	0.77 (0.42-1.41)	0.400		
**Histology**				
Non-mucinous	1 (reference)			
Mucinous	0.84 (0.46-1.55)	0.581		
**Primary Differentiation**				
Well/moderately	1 (reference)		1 (reference)	
Poorly/undifferentiated	1.77 (0.94-3.34)	**0.076**	1.59 (0.83-3.03)	0.163
**T stage**				
T1-2	1 (reference)			
T3-4	0.64 (0.35-1.17)	0.144		
**N stage**				
N0	1 (reference)		1 (reference)	
N1-2	2.13 (1.14-3.98)	**0.018**	1.59 (0.82-3.05)	0.168
**M stage**				
M0	1 (reference)		1 (reference)	
M1	1.72 (1.12-2.64)	**0.013**	1.61 (1.04-2.49)	**0.033**
**TNM stage**				
I	1 (reference)			
II	2.27 (0.60-8.56)	0.226		
III	2.46 (0.69-8.83)	0.167		
IV	4.30 (1.28-14.50)	**0.019**		
**Nerve invasion**				
No	1 (reference)			
Yes	1.47 (0.46-4.77)	0.518		
**Vascular invasion**				
No	1 (reference)			

Yes	1.77 (0.82-3.83)	0.145		
**Lymph nodes examined**				
＜12	1 (reference)			
⩾12	0.65 (0.23-1.82)	0.414		
**MMR status**				
pMMR	1 (reference)			
dMMR	1.50 (0.67-3.38)	0.326		
**The density of CXCR6+TAMs**				
Low	1 (reference)		1 (reference)	
High	2.61 (1.33-5.11)	**0.005**	2.07 (1.03-4.17)	**0.041**

CEA, carcinoembryonic antigen; MMR, mismatch repair.

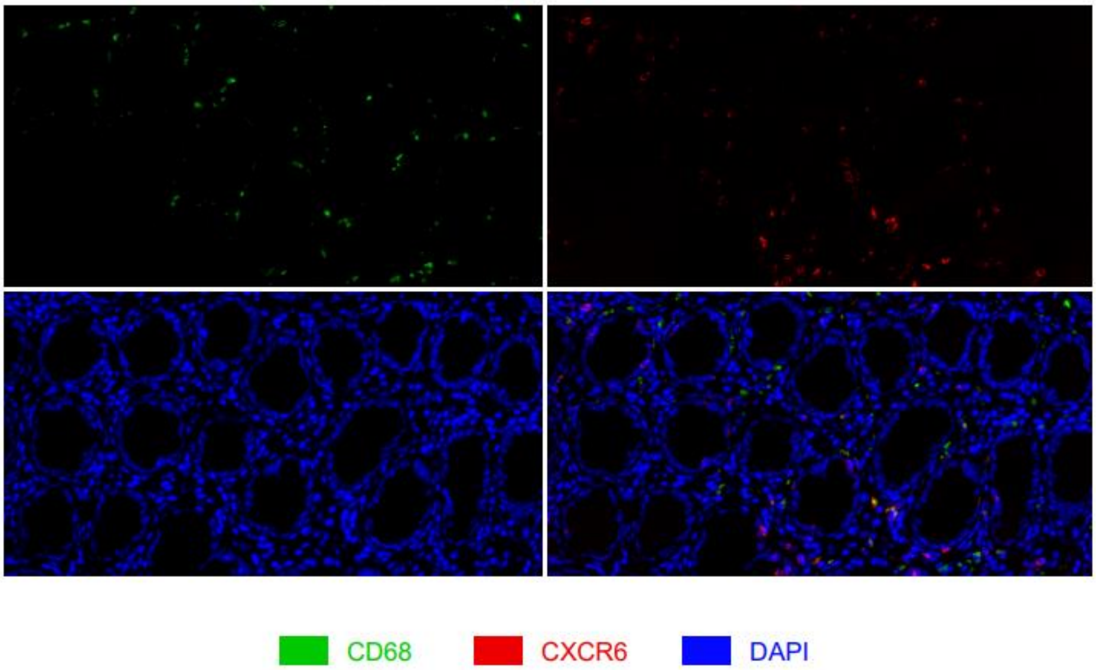

Figure S1. Representative images of CXCR6+ TAMs in peritumor specimens.

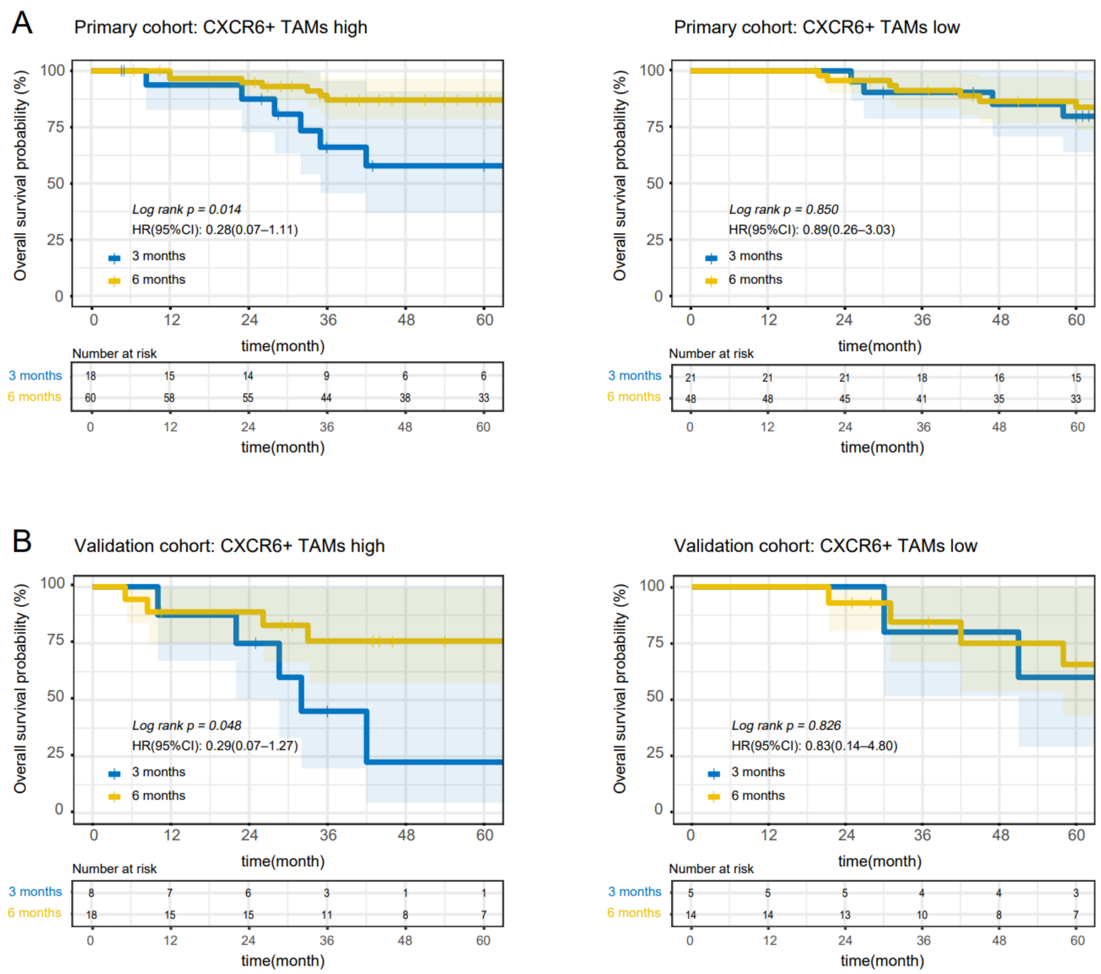

Figure S2. High CXCR6+ TAMs infiltration is associated with better response to 6-month adjuvant chemotherapy in high-risk II stage and III stage CC patients. (A) Overall survival curves of patients with high and low CXCR6+ TAMs from primary cohort. (B) Overall survival curves of patients with high and low CXCR6+ TAMs from validation cohort.
